# Eating Wood

**Published:** 1875-03

**Authors:** 


### EATING WOOD.

Wood is not eatable; it is not digestible; yet it is very largely masticated. You may watch those who step out of any hotel or restaurant, and you will observe that nine-tenths are chewing wood. On the desk, at most public dining-rooms, you will find bundles of wood, which are free to all guests, and of which the majority avail themselves. Wood is therefore unquestionably an article of daily diet with a good many people, and is more thoroughly chewed than anything on the bill of fare.

It was a good idea, that of cutting up poplar and basswood into clean splinters, to be used as toothpicks. If everybody would pass one of these thin strips of wood between all their teeth, every time after eating, their teeth would last much longer, because the food which, by decomposing in contact with the teeth, dissolves and destroys their integrity, would thus be removed before becoming alike offensive and injurious. This is the object of the toothpick, and the habit of thus using it is clearly a habit in the interest of neatness and good breeding.

But the practice of carrying these splinters for hours, or even minutes, in the mouth is thoroughly offensive to good taste. Their use is a private act, and hardly anything can be more objectionable or annoying to the spectator than this nearly universal toothpick exhibition. We have seen men and women enter the drawing-room from the dinner-table, and publicly employ the wooden splinters, with varying energy, for an hour or so, unconscious that they were doing a very unclean thing.

There is another feature of this business which it is worth while to consider. In carrying these fragments of wood in the mouth, they are almost certain to become ground up and divided, and the result is that woody particles are swallowed. Nature manages to take care of foreign substances, when such are swallowed, and either to provide for their exit or for their retention with the smallest possible injury, as we have shown in our recent article on indigestible substances; but this is no reason why we should unnecessarily burden the stomach with useless and indigestible trash. We certainly impose much unnecessary labor upon the digestive machine when we thus constantly "poke sticks" at it. It is unfair to the honest stomach, from which we demand support, to treat it as we do. It is bad enough to feed it hastily, as is our custom, with much "that is not bread," and to compel it to labor under the burden of many things which clearly satisfy not; but it is abominable to thus unnecessarily irritate it with woody fibres. Can we not employ these minute splinters which cost so little, and which are really so useful when properly used, in such a manner that they will neither offend good manners nor engender dyspepsia?

---

Beauty, wit, high birth, vigor of health, desert in service, love, friendship, charity, are subject all to envy and calumny.

---

Some friends put us out of conceit with friendship, just as some religious people put us out of conceit with religion.

